# Drought alleviation efficacy of a galactose rich polysaccharide isolated from endophytic *Mucor* sp. HELF2: A case study on rice plant

**DOI:** 10.3389/fmicb.2022.1064055

**Published:** 2023-01-26

**Authors:** Hiran Kanti Santra, Debdulal Banerjee

**Affiliations:** Microbiology and Microbial Biotechnology Laboratory, Department of Botany and Forestry, Vidyasagar University, Midnapore, West Bengal, India

**Keywords:** optimisation, drought stress alleviation, *Mucor* sp. HELF2, endophyte, hetero polysaccharide

## Abstract

Endophytes play a vital role in plant growth under biotic and abiotic stress conditions. In the present investigation, a Galactose-Rich Heteropolysaccharide (GRH) with a molecular weight of 2.98 × 10^5^ Da was isolated from endophytic *Mucor* sp. HELF2, a symbiont of the East Indian screw tree *Helicteres isora*. OVAT (One Variable at A Time) experiment coupled with RSM (Response Surface Methodology) study exhibited 1.5-fold enhanced GRH production (20.10 g L^−1^) in supplemented potato dextrose broth at a pH of 7.05 after 7.5 days of fermentation in 26°C. GRH has alleviated drought stress (polyethylene glycol induced) in rice seedlings (*Oryza sativa* ssp. indica MTU 7093 swarna) by improving its physicochemical parameters. It has been revealed that spray with a 50-ppm dosage of GRH exhibited an improvement of 1.58, 2.38, 3, and 4 times in relative water contents and fresh weight of the tissues, root length, and shoot length of the rice seedlings, respectively “in comparison to the control”. Moreover, the soluble sugars, prolines, and chlorophyll contents of the treated rice seedlings were increased upto 3.5 (0.7 ± 0.05 mg/g fresh weight), 3.89 (0.57 ± 0.03 mg/g fresh weight), and 2.32 (1,119 ± 70.8 μg/gm of fresh weight) fold respectively, whereas malondialdehyde contents decreased up to 6 times. The enzymatic antioxidant parameters like peroxidase and superoxide dismutase and catalase activity of the 50 ppm GRH treated seedlings were found to be elevated 1.8 (720 ± 53 unit/gm/min fresh weight), 1.34 (75.34 ± 4.8 unit/gm/min fresh weight), and up to 3 (100 ppm treatment for catalase – 54.78 ± 2.91 unit/gm/min fresh weight) fold, respectively. In this context, the present outcomes contribute to the development of novel strategies to ameliorate drought stress and could fortify the agro-economy of India.

## Introduction

The foundation of the global food economy is agriculture, and in a nation like India, Gross Domestic Production (GDP) is heavily reliant on the agrarian model. Crop loss due to biotic and abiotic stressors is a widespread issue that requires effective management techniques to keep the agroecosystem in good shape. Biotic stress-related issues can be addressed with a variety of chemical formulations, such as pesticides, herbicides, fungicides, and biological techniques, such as biological control agents (BCA) and plant growth promotors (PGPs), but abiotic stress management strategies have received scant attention. As a result, issues with salt and drought stress are severely impeding crop development and yield, with dryness being the most detrimental. Agriculture production is significantly declining globally (Chen et al., 2017). The problem is getting worse as a result of cases of global warming and water scarcity. The agrarian economy is struggling with production-related problems as well as severe financial constraints (Vurukonda et al., 2016). According to reports, to fulfil this ambitious goal by 2050, food production must increase by up to 60 to 110 percent. Drought-related challenges must be immediately resolved (Naumann et al., 2018; Dey et al., 2019; Paglia and Parker, 2021). Therefore, it is urgent to discover a new, long-lasting solution to this global dilemma (Coleman-Derr and Tringe, 2014). One of the initial answers to that problem is to create stress-resistant varieties, but doing so takes time, is rigorous, species-specific, and is expensive (Santra and Banerjee, 2022a). One approach might be to cultivate crops on reclaimed drought-affected land while using foliar plant growth-promoting/stress-resisting chemicals. Rhizobacteria that promote plant growth have already been evaluated for this purpose, and less-studied endophyte or endophytic fungal or bacterial polysaccharides are currently showing promise in this field (Chen et al., 2017; Sun et al., 2020a,b).

Endophytes are ubiquitous in occurrence and are procured from nearly all plants and plant parts studied to date across the globe (Coleman-Derr and Tringe, 2014; Chatterjee et al., 2022). The symbionts of plant tissues known as endophytes help the plant grow and give tolerance in challenging conditions. Due to their horizontal gene transfer as a result of the co-evolution of the host and microorganisms, they share crucial genes of essential metabolomes (Santra et al., 2022; Santra and Banerjee, 2022b; Santra and Banerjee, 2022c). They have a reputation for being bioactive chemical mines that are simple to access using contemporary biotechnological methods. Plant growth-promoting endophytic bacteria and fungi reside on different internal plant tissues and organs, i.e., in stems, roots, flowers, leaves, fruits, and seeds. Endophytes have recently drawn attention because they are an effective tool for teaching plants to tolerate lethal abiotic stressors like drought, salt, and heavy metal toxicity (Cherif et al., 2015; Mesa et al., 2015; Pinedo et al., 2015; Constantin et al., 2019; Moghaddam et al., 2021) through adopting various mechanisms. The two most prevalent and important methods of building stress resistance are decreasing the levels of the key gaseous hormone ethylene through the activity of ACC (1-aminocyclopropane−1-carboxylate) deaminase and increasing the content of prolines in the tissues (Blaha et al., 2006; Gamalero and Bernard, 2012; Marasco et al., 2012). In addition to these, accumulation of siderophores and osmolytes, increased antioxidant and photosynthetic rates, synthesis of phytohormones and organic acids, and emission of volatile organic compounds are other important mechanisms used by endophytes to increase host plant abiotic stress tolerance (Tiwari et al., 2016; Vurukonda et al., 2016). Recent reports include that microbial symbionts or fungal endophytes are the co-evolution partners of green plants and promote habitat-specific stress tolerance in host plants (; Rodriguez and Redman, 2008; Redman et al., 2011). A special type of long-chain polymeric secondary metabolite called exopolysaccharides (EPS) from endophytic sources holds immense agricultural utility especially in ameliorating drought and salt stress (Nadeem et al., 2014; Rolli et al., 2015).

In the current study, exopolysaccharide was extracted from the endophytic fungus *Mucor* sp. HELF2 (isolated from *Helicteres isora* flowers). The EPS was galactose-rich heteropolysaccharide (GRH) in composition. GRH was found to be effective in reducing drought stress conditions when applied to the foliar parts of the rice seedling *Oryza sativa* ssp. indica MTU 7093 swarna. GRH production by HELF2 was optimised by adopting statistical modelling using Minitab and the predicted model led to an enhancement of 1.5 times exopolysaccharide (GRH) production under optimised fermentation conditions. The application of 50 ppm GRH was discovered to be the most efficient dosage, and the physical/biochemical traits of the treated plants were discovered to be higher than those of the untreated ones. Root and shoot length, fresh weight, enzymatic antioxidant profiles, and proline contents were improved remarkably after treatment. The membrane damage caused by lipid peroxidation was also minimised when GRH was applied *in vivo*. MDA content was reduced and SOD, CAT, and POD values were elevated. The current study illuminates the agricultural potential of endophytic exopolysaccharide, which has the potential to expand the field of sustainable development and improve the agro-economy of our nation’s indigenous population.

## Materials and methods

### Isolation and identification of GRH-producing endophytic fungi

*Mucor* sp. HELF2 was isolated as an endophyte from the flower of an ethnomedicinally valuable plant *Helicteres isora* collected from forests in the East Singbhum district, Jharkhand, West Bengal, India, and stored, maintained on PDA slants and Petri plates at 4 ± 2°C and 25 ± 2°C, respectively. In brief, plant parts were thoroughly washed by running tap water for 5 min, sodium hypochlorite (2–10%) for 2 min, and hydrogen peroxide (2%) for 1 min, respectively, and explants were incubated on water agar plates at 27°C on biological oxygen demand incubator for endophyte isolation. Water agar plates were supplemented with antibiotics (streptomycin and tetracycline- 50 mg L^−1^) to avoid bacterial endophytes. The effectiveness of this sterilisation and isolation process was cross-checked by the explant imprintation technique described by Schulz et al. (1993). In brief, the aliquots used for explant sterilisation were spread on a water agar medium and incubated under the same conditions. After, 3–5 days of incubation, fungal hyphae emerged from the tissues and they were transferred to PDA plates for optimum growth (Schulz et al., 1993). Emerging fungal hyphal tips were transferred to PDA (Potato Dextrose Agar) medium and morphology (both macroscopic and microscopic) of the fungal isolate was recorded using light (Primo Star, Zeiss, Germany) and stereo microscope (Stemi 508, Zeiss, Germany).

The organism was identified by rDNA-based molecular technique as there was no reproductive structure produced by the endophytic fungi even in a medium with carnated leaves. In brief, genomic DNA of the fungal isolate was obtained (using DNeasy Plant Minikit-Qiagen, Germany) and a polymerase chain reaction was performed using the two universal primers named ITS1 (5’-TCCGTAGGTGAACCTTGCGG-3′) and ITS4 (5’-TCCTCCGCTTATTGATATGC-3′; Laich and Andrade, 2016; Landum et al., 2016). The PCR products were separated using 1% agarose gel in 1X TAE buffer (90 mM Tris-acetate and 2 nM EDTA, pH 8.0), stained with ethidium bromide (0.5 μg mL^−1^), and documented using BIO-RAD Gel Doc EZ imager version 5.1 (United States). PCR products were sent for direct bi-directional sequencing using ABI 3730xl Genetic Analyzer (Applied Biosystems, United States) to Bioserve Biotechnologies (India) Pvt. Ltd., A Repro Cell Company, Hyderabad, India. The obtained consensus sequence of 620 bp was used for further study. Sequences were submitted to GenBank and were compared to the GenBank database using BLAST. Fifteen sequences along with HELF2 were selected and aligned using the multiple alignment software program Clustal W and the phylogenetic tree was prepared using MEGA 11 (Tamura et al., 2021).

### Production of GRH and optimisation of culture conditions by OVAT technique

Endophytic fungi were grown in different 250 mL Erlenmeyer flasks with 50 mL potato dextrose broth in a shaker incubator at 120 rpm for 8 days. An initial medium pH of 6 and a medium temperature of 28°C were maintained.

To detect the optimum culture conditions for the maximum production of GRH, fungi were grown in varying fermentation times (4–10 days), then in different medium pH (5.5–7.9), and then at varying incubation temperatures (20–30°C) in separate Erlenmeyer flasks with separate PDB medium. To find out the requirement of additional nutrients for maximum GRH production and mycelial growth, various carbon sources (5 g%, w/v of fructose, glucose, maltose, starch, rhamnose, raffinose, glycerol), various organic and inorganic nitrogen sources (0.4 g% w/v of peptone, ammonium nitrate, urea, ammonium chloride, glycine and yeast extract) in different Erlenmeyer flasks were used with PDB as the basal medium. After the finalisation of the additional carbon and nitrogen sources, their optimum concentration was confirmed by using different concentrations of these products on a PDB medium and the respective biomass and GRH amounts were calculated. A variety of ionic salts (0.1 g%, w/v of MgCl_2_, FeCl_3_, KCl, NaCl) and phosphate sources (0.1 g%, w/v including NaH_2_PO_4_, K_2_HPO_4_, KH_2_PO_4_) were analysed separately to detect their role in fungal biomass and GRH production (Mahapatra and Banerjee, 2013, 2016).

To detect the O_2_ requirement, fungi were grown with different medium volumes in 250 mL Erlenmeyer flasks. Headspace volume, medium volume, total volume, and medium depth in flask culture were measured for the indirect measurement of the organism’s O_2_ requirement (Wonglumsom et al., 2000).

### BBD based optimisation

Further optimisation was performed with the RSM (Response surface methodology). The investigational design was a Box–Behnken experimental setup with the four most important factors obtained from the OVAT system. The four independent factors had three different levels (−1, 0, and + 1) each for the experiment. GRH production was set to a second-order polynomial equation by the means of multiple regression techniques. The model involving the most significant factors was derived. The system performance follows the subsequent second-order polynomial equation: Y = β_0_+ Σβ_i_X_i_ + Σβ_ij_X_i_X_j_ + Σβ_ii_X^2^_i_, where Y is the predicted response or dependent variable, x_i_ and x_j_ are independent factors, β_0_ is the intercept of the regression equation, β_i_ is the linear coefficient, β_ii_ is the quadratic coefficient and β_ij_ is the interaction coefficient (Mahapatra and Banerjee, 2013, 2016).

### Estimation of GRH

Fungal biomass was separated from the culture extract by centrifugation at 10,000 rpm. Mycelial biomass was dried at 55°C for 24 h and weighed. The supernatant was concentrated in a rotary evaporator under low pressure at 40°C. Chilled absolute ethanol was added to the concentrated supernatant (5:1 v/v), mixed thoroughly, and kept for 24 h at freezing conditions finally, the recovery of viscous precipitate was done by centrifugation at 10,000 rpm for 10 min. The recovered polysaccharide was dialyzed in a cellulose membrane (MW cut off 10,000) against distilled water for 24 h. It was tested for sugar and protein contents following the methods of Dubois et al. (1956) and Lowry (1951) with glucose and bovine serum albumin as the standard. Obtained EPS solution was concentrated in a rotary evaporator under low pressure at 40°C for characterisation.

### Characterisation of GRH

GRH was purified by gel chromatographic technique using a Sepharose-6B gel filtration column (65 × 2 cm) and average molecular weight was determined following the methods of Mahapatra and Banerjee (2013). The dried polysaccharide was subjected to characterisation using GC–MS with some pre-treatments (Proestos et al., 2006). Using a water bath at 70°C for 15 min, 100 mg of dried exopolysaccharide was combined with 1 mL MeOH, 20 μl ribitol (which serves as an internal standard), and 20 μl nor-leucine. The entire mixture was then centrifuged for 5 min at 10,000 rpm, and the supernatant was immediately dried and dissolved in 20 μl of methoxy-amine HCL for 120 min at 37°C. The final 1 μl of the derivatized EPS sample was loaded onto the GC–MS for analysis of monosaccharide composition after 40 μl of TMS (Trimethyl siloxane) had been added. The instrument was set up with a 30 m × 0.25 mm DB-5 Ultra Inert column. With a split ratio of 25:1, the inlet temperature was 230°C, and the MS transfer line temperature was 250°C. A constant flow mode was used for the column’s flow, which was 1.3 mL min^−1^ with an average linear starting velocity of 39 cm sec^−1^. Helium was used as the carrier gas, and ZB − 1701 served as the guard column. The program was isothermal, holding at 70°C for 5 min, increasing by 10°C per minute to 180°C, holding for 2 min, increasing by 10°C per minute again to 220°C, holding for 1 min, and finally ramping up by 2.5°C per minute. Up to 265°C with a 1-min hold, then a ramp up to 285°C with a 1-min hold, and finally a climb of 1°C per min up to 290°C with a 0.6-min hold. The mass spectrum was obtained in scan mode from 40 to 650 amu with a detection threshold of 100 ion counts while the detector was in positive ion mode. Appropriate configurations (D-dextrorotatory and L-laevorotatory form) of the sugars were identified by matching them with the NIST library. To detect the sugar linkages, procedures of Ciucanu and Kerek (1984) and Das et al. (2009) were adopted using the GC–MS equipment.

### Exopolysaccharide (GRH)-mediated plant growth promotion under drought stress

Initially, healthy and disease-free rice seeds (*O. sativa* ssp. indica MTU-7093 Swarna) were surface sterilised with a series of surface disinfectants: sodium hypochlorite (2.5%) for 20 min, deionised double-distilled water (3–5 times thoroughly) and then soaked in water for germination followed by storing at 22°C for 72–96 h. Uniformly seeds were transferred to a hydroponics box supplemented with Hoagland solution and were replaced at an interval of 3–4 days (Chen et al., 2011). Seedlings reaching an age of 15–20 days were divided into four separate groups with the control group (un-inoculated water), 20 ppm, 50 ppm, and 100 ppm GRH application, respectively, at a frequency of 3 times a day (morning, noon, and afternoon) for 45 days. Simultaneously, 20% polyethylene glycol (PEG)-6,000 (for 7 days) was mixed with a hydroponic solution as a drought-inducing component, and all the biochemical tests were performed from drought-induced seedlings. The fresh weight of rice seedlings was measured and leaves were stored at −20°C for further biochemical estimations.

The relative water content (RWC) of the treated and control plants were calculated in percentage following the method of Arndt et al. (2015). Fresh leaves were plucked and fresh tissue weight (FW) was measured, then immersed in a 50 mL tube with distilled water, and placed in the dark at 4°C for 20 h. Further, the leaves were dried with filter paper and again weighed for turgid weight (TW) calculation. Lastly, the same leaves were incubated at 80°C for a period of 72 h and dry weight (DW) was measured immediately. Relative water content was calculated by the formula RWC (%) = (FW−DW)/ (TW−DW).

The chlorophyll content (mg g^−1^ of fresh weight) of the fresh leaves was measured according to the modified formula of Lichtenthaler and Wellburn (1983). Firstly, fresh leaves (0.5 g fresh weight) were split into small pieces and immediately dissolved in 50 mL methanol (80% v/v), covered with black paper, or kept in dark conditions for 24–36 h at 28–30°C. Centrifugation was performed and the supernatant was estimated (645 nm and 653 nm) for chlorophyll contents. Chlorophyll content (mg L^−1^ FW) = 8.05 A_653_ + 20.29 A_645_.

To calculate the proline contents of the seedling methods proposed by Bates et al. (1973) were adopted. Leaves (0.5 g fresh weight) were split into small pieces and put in a test tube. Further treatment was done by mixing with 5 mL of 3% sulfosalicylic acid, incubated in a water bath for 10 min and 2 mL of the supernatant was mixed thoroughly with 2 mL of acetic acid, and 3 mL of 2.5% ninhydrin. Finally, the mixture was incubated in the water bath for a time period of 40 min^−1^ h and extracted using 4 mL methylbenzene, optical density was measured at 520 nm and compared with a proline standard curve.

Soluble sugar contents (in terms of mg g^−1^ fresh weight) were measured by the method of Watanabe et al. (2000). Fresh leaves (0.2 g fresh weight) were crushed in 80% v/v ethanol (10 mL) and centrifuged at 8000 g for 10 min at 4°C. We mixed 1 mL of supernatant thoroughly with 3 mL of anthrone reagent followed by heating at 100°C for 10–12 min, which was stopped by rapid cooling them on the ice. Finally, at 620 nm absorbance was estimated using glucose as a standard.

Malondialdehyde content (nmol g^−1^ fresh weight) was reported according to the method of Del Buono et al. (2011) 0.5 gm of fresh leaves were homogenised in 5% (w/v) trichloroacetic acid (TCA), centrifuged at 12000 g for a time period of approximately 15–20 min and then the supernatant was mixed with 5 mL of 0.5% thiobarbituric acid (TBA)-prepared with 20% TCA followed by incubation for 25 min and cooling at 100°C and room temperature, respectively. Finally, after centrifugation (7,500 g for 5 min), the supernatant was measured for its absorbance at 450, 532, and 600 nm. The amount of MDA was calculated by the following formula MDA content (nmol g^−1^) = 6.45 (A_532_-A_600_)−0.56A_450_.

The methods of Lei et al. (2015) were followed for the measurement of peroxidase activity. The system contained multiple chemicals; 2.9 mL of 0.05 M phosphate buffer, 0.5 mL of 2% H_2_O_2_, 0.1 mL of 2% guaiacol, and also 0.1 mL of crude enzyme extract followed by the absorbance measurement at 470 nm. Lastly, POD activity was calculated as an amount of guaiacol oxidised per minute in nanomoles per minute per mg of protein. At the end of the reaction, the absorbance was measured at 470 nm. POD activity was defined as the amount of guaiacol oxidised per minute, and was expressed as nanomoles per minute per mg of protein.

Catalase activity was calculated following the protocols of Lei et al. (2015). O.1 mL H_2_O_2_ (2%) and 2 mL phosphate buffer (50 mM-pH 7.0) were mixed and the whole reaction was initiated by the addition of 0.1 mL of crude enzyme extract. Finally, the catalase activity was measured (at 240 nm) in terms of the decrease of values of H_2_O_2_ per minute, as nanomoles/min/gm of protein.

Superoxide dismutase activity was assayed following the protocols of Lei et al. (2015). The whole system contained a series of valuable freshly prepared reagents; 1.5 mL of 0.05 M phosphate buffer, 0.3 mL of 130 mM methionine solution, 0.3 mL of 750 μM nitroblue tetrazolium solution, 0.3 mL of 100 μM EDTA -Na_2_ solution, 0.3 mL of 20 μM lactochrome solution, 0.5 mL of distilled water and finally 0.1 mL of crude enzyme extract. The complete reaction was initiated at 4000 Lx of illumination for a constant 20 min with no interruption. The control set comprises the same set of reagents and illumination but with no crude enzyme extract, rather replaced with a phosphate buffer. The third setup of control contains only phosphate buffer followed by incubation in dark conditions for the same time period of 20 min. Finally, after the completion of the reaction, the absorbance was estimated at 560 nm of wavelength. One unit of SOD activity was defined as the amount of enzyme which inhibits NBT reduction by 50%, also the results were expressed as unit/mg protein.

### Statistical analysis

All experiments were performed in triplicate and the results are presented as means ± standard errors (SE). Data were analysed by Prism GraphPad version 9.2.0 (332) software (San Diego, California, United States). BBD experiments were done in Minitab (version 20.2).

## Results

### Identification of the isolate

**Figure 1:**
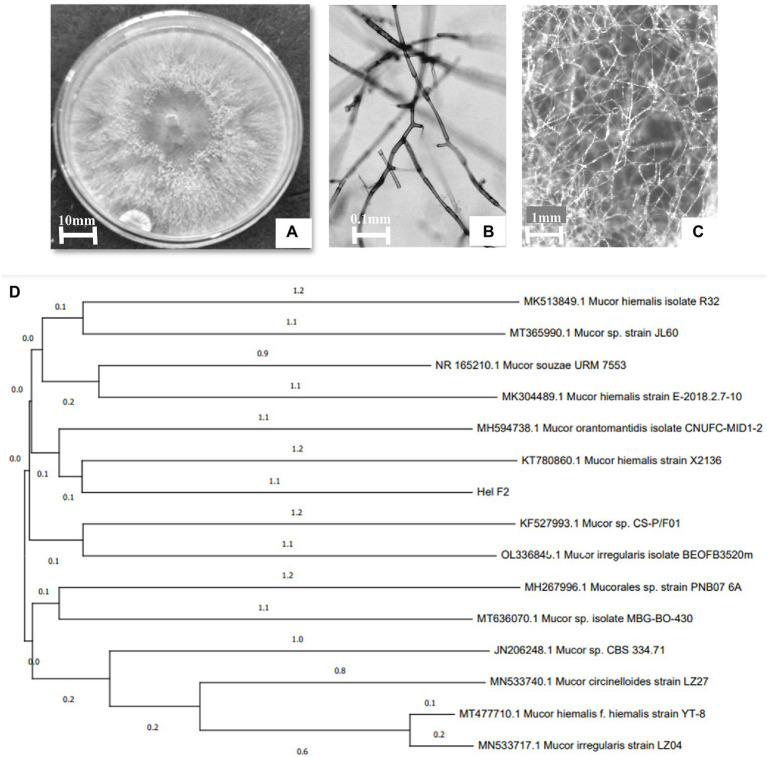
**(A)** 8-day old culture of HELF2 grown on PDA medium. Sterile hyphal aggregation without any sexual or asexual reproductive structures seen under a light **(B)** and stereo **(C)** microscope. **(D)** Phylogenetic tree shows the relationship of endophytic *Mucor* sp. HELF2 with other *Mucor* sp. strains.

The organism had enormously intertwining hyphae and septate hyaline whitish mycelium, and it lacked any sexual or asexual reproductive structures (Figures 1A–C). rDNA sequence data of the isolate was deposited in GenBank (ON146358). A BLAST search of the earlier existing database indicates a close genetic connection with other species of Mucor and the evolutionary history of the endophytic fungal isolate HELF2 was included using the neighbour-joining method (Saitou and Nei, 1987). The most appropriate phylogenetic tree with a total branch length of 0.00649585 is represented in Figure 1D. The tree was constructed to scale with branch lengths in similar units as those of the evolutionary distances used to infer the phylogenetic tree. The evolutionary distances between the species were analysed using the maximum composite likelihood method (Tamura et al., 2021) and were in the units of the number of base substitutions per site. Gaps and missing data were removed from the dataset. There was a total of 620 nucleotides in the final dataset.

### Optimisation of GRH production

*Mucor* sp. HELF2 was grown on a 250 mL Erlenmeyer flask in submerged condition for 10 days and the highest production of fungal GRH and biomass was detected after 8 days of fermentation (Table 1). A temperature of 26°C and a medium pH of 7.1 was found to be the most suitable one for GRH production. Glucose and peptone at a concentration of 11 g L^−1^ and 5.5 g L^−1^ were found to be the most appropriate ones for maximum GRH yield (Table 1). NaCl at a concentration of 0.1 g L^−1^ was the most effective salt (or source of metal ions) for GRH production. The detailed effect of different parameters on biomass and GRH production is summarised in Table 1.

**Table 1:** Effect of different fermentation influencing physical conditions and chemical supplements, on biomass and Galactose Rich Heteropolysaccharide production by endophytic fungi *Mucor* sp. HELF2 in submerged fermentation conditions.

Parameters tested	Effectors	The concentration of the effectors (g L^−1^)	Biomass (g L^−1^)	GRH (g L^−1^)
Fermentation time (h)	4 days	–	6.1 ± 0.04a	13.42 ± 0.03a
	6 days	–	5.99 ± 0.01a	13.96 ± 0.04b
	8 days	–	6.96 ± 0.8b	14.44 ± 0.03c
	10 days	–	5.76 ± 0.02c	13.11 ± 0.05a
Fermentation temperature (°C)	22	–	7.02 ± 0.03a	14.48 ± 0.02a
	24	–	7.18 ± 0.05a	15.51 ± 0.07b
	26	–	7.47 ± 0.01b	16.51 ± 0.08c
	28	–	6.96 ± 0.08a	16.11 ± 0.09b
Initial medium pH	6.9	–	7.7 ± 0.07a	17.51 ± 0.01a
	7.1	–	7.81 ± 0.06b	17.71 ± 0.04a
	7.3	–	7.73 ± 0.01a	17.41 ± 0.01a
	7.5	–	7.6 ± 0.05c	16.91 ± 0.04b
Additional carbon sources	Fructose	–	7.88 ± 0.03a	18.11 ± 0.01a
	Glucose	–	7.97 ± 0.05b	19.12 ± 0.05b
	Maltose	–	7.9 ± 0.06a	19.02 ± 0.03b
	Starch	–	7.82 ± 0.09c	18.9 ± 0.03c
	Rhamnose	–	7.87 ± 0.01a	18.93 ± 0.02c
	Raffinose	–	7.79 ± 0.02c	18.82 ± 0.02c
	Glycerol	–	7.82 ± 0.06c	18.51 ± 0.03d
Additional nitrogen sources	Peptone	0.4	8.71 ± 0.06a	19.86 ± 0.03a
	(NH_4_)_2_SO_4_	0.4	7.99 ± 0.02b	19.28 ± 0.04b
	NH_4_NO_3_	0.4	8.1 ± 0.07b	19.41 ± 0.04c
	Urea	0.4	8.23 ± 0.04c	19.52 ± 0.05c
	NH_4_Cl	0.4	8.16 ± 0.02b	19.7 ± 0.06d
	Glycine	0.4	8.2 ± 0.01c	19.74 ± 0.05d
	Yeast Extract	0.4	8.41 ± 0.09d	19.12 ± 0.06b
Glucose concentration	Glucose	7	9.18 ± 0.05a	19.9 ± 0.07a
		9	9.27 ± 0.06b	20.18 ± 0.02b
		11	9.47 ± 0.05c	20.23 ± 0.04b
		13	9.3 ± 0.06b	20.12 ± 0.03b
Peptone concentration	Peptone	4.5	9.59 ± 0.07a	20.24 ± 0.03a
		5	9.53 ± 0.08a	20.32 ± 0.04a
		5.5	9.7 ± 0.01b	20.5 ± 0.05b
		6	9.49 ± 0.06a	20.42 ± 0.02b
Different metal ions	MgCl_2_	0.1	9.3 ± 0.01a	20.52 ± 0.06a
	FeCl_3_	0.1	9.39 ± 0.06b	20.62 ± 0.03b
	KCl	0.1	9.51 ± 0.07c	20.81 ± 0.04c
	NaCl	0.1	9.8 ± 0.04d	20.92 ± 0.07d
	NaH_2_PO_4_	0.1	9.48 ± 0.03c	20.71 ± 0.08b
	K_2_HPO_4_	0.1	9.6 ± 0.07e	20.51 ± 0.09a
	KH_2_PO_4_	0.1	9.57 ± 0.08e	20.42 ± 0.10e

One-way ANOVA (Tukey’s Multiple Comparison test) was performed to check the potential statistical differences in the case of the biomass (g L^−1^) and GRH production (g L^−1^) in different fermentation conditions (incubation time, temperature, the concentration of sugar and nitrogen sources, etc.). There were valid statistical differences in most of the cases (*p* < 0.05), the different letters a, b, c, d, e, and f indicate a significant difference, and the same letter at two positions indicates no statistical differences.

The amount of dissolved oxygen in the fermentation medium affects EPS production. It depends on the medium volume, the headspace of the medium, and the medium depth. A medium volume of 75 mL in a 320 mL Erlenmeyer flask with 245 mL of headspace volume and 2.1 cm of medium depth and 2.53 cm of surface area was found to be the criteria for optimum GRH production (Table 2).

**Table 2:** Effect of medium volume, headspace volume, and medium depth on the dissolved oxygen level in the fermentation medium and their effect on GRH and biomass production.

Medium volume (mL)	The total volume of the flask (mL)	Headspace volume (mL)	Medium depth (cm)	Surface area (cm)	Biomass (g L^−1^)	GRH (g L^−1^)
25	320	295	1	2.5	11.66 ± 0.18a	21.01 ± 0.021a_1_
50	320	270	1.5	2.58	11.8 ± 0.75a	21.42 ± 0.049b_1_
75	320	245	2	2.53	12.99 ± 0.43b	22.04 ± 0.089c_1_
100	320	220	2.5	2.41	11.69 ± 0.64a	21.28 ± 0.071a_1_

One-way ANOVA (Tukey’s Multiple Comparison test) was performed to check the potential statistical differences between the data (column-wise) of biomass (g L^−1^), and GRH production (g L^−1^) in different medium volumes (mL). There was a valid statistical difference in most of the cases. Different letters (a, b for biomass and a_1_, b_1_, c_1_ for GRH yield) indicates valid statistical differences (*P* < 0.05).

**Figure 2:**
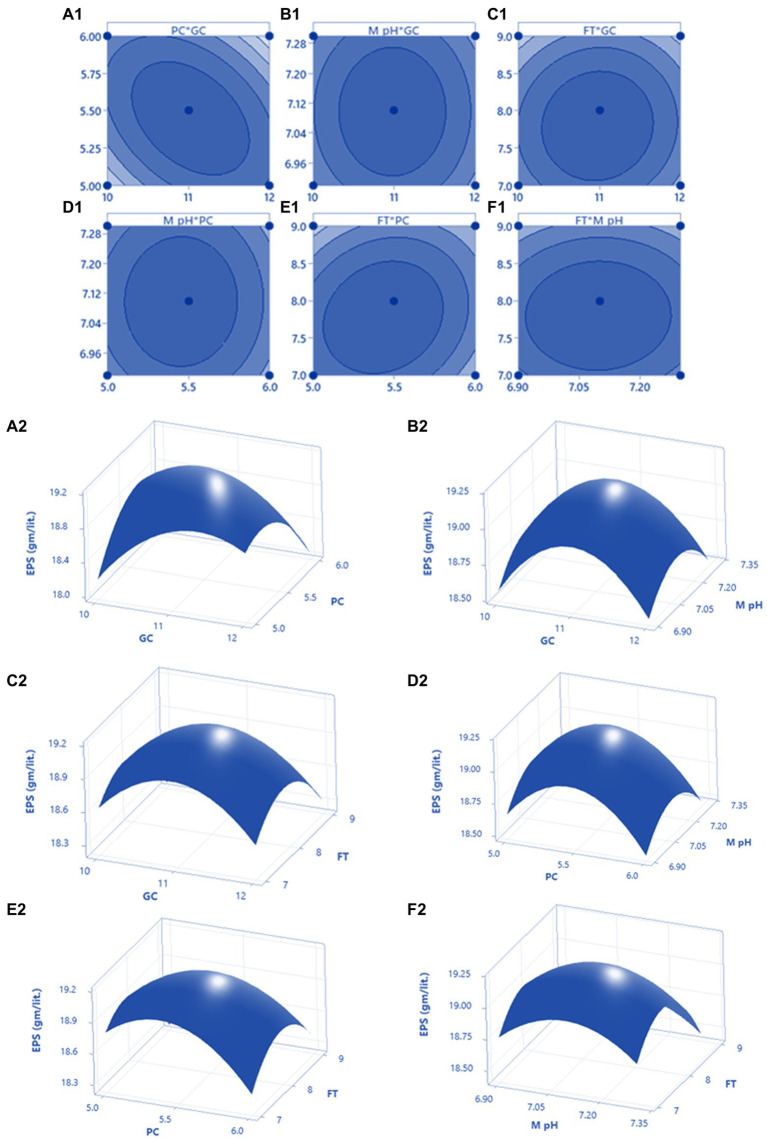
The 3D plot with 2D projection and contour plot showing the most important interactions of factors in RSM optimization of GRH production by HELF2. **(A1,A2)** between peptone concentration (PC) vs. glucose concentration (GC) at fermentation time (FT) 8 days and medium pH 7.1 (MpH); **(B1,B2)** between MpH (7.1) and GC (11 g L^−1^) at FT (8 days) and PC (5.5 g L^−1^); **(C1,C2)** between FT (8 days) and GC (11 g L^−1^) at PC (5.5 g L^−1^) and MpH (7.1); **(D1,D2)** between MpH (7.1) and PC (5.5 g L^−1^) at FT (8 days) and GC (11 g L^−1^); **(E1,E2)** between FT (8 days) and PC (5.5 g L^−1^) at MpH (7.1) and GC (11 g L^−1^); **(F1,F2)** between FT (8 days) and MpH (7.1) at GC (11 g L^−1^) and PC (5.5 g L^−1^).

After OVAT optimisation RSM was adopted using a three-level Box Behnken Design. The most important four factors (glucose concentration, peptone concentration, medium pH, and fermentation time) with five replicates at the center points were established as a model for analysis of GRH production. The experimental design with variable predicted and measured values of GRH was presented in Table 3. Maximum GRH production was noted at the five replicated center points. The predicted response Y for GRH production by *Mucor* sp. HELF2 was described as coded factors in the following equation Y_GRH_ = 5.9810–0.168024x_1_–0.39671x_2_-0.09103x_3_-0.37144x_4_-0.25686x_1_x_2_–0.03737x_1_x_3_ + 0.09369x_1_x_4_–0.0367 x_2_x_3_ + 0.32736 x_2_x_4_–0.0337 x_3_x_4−_0.65663 x^2^_1_-0.6395 x^2^_2−_0.50414 x^2^_3_–0.85426 x^2^_4_. Here Y_GRH_ is the predicted GRH yield and x_1_, x_2_, x_3_, and x_4_ are the four coded factors of glucose concentration, peptone concentration, medium pH, and fermentation time (day) respectively. A regression analysis with detailed statistical data related to the experiment is presented in Table 4. The F-test data of 1994.082 proved that the model was significant. The adjusted determinant coefficient (R^2^ Adj) was found to be 0.9998 which represents that there is a high degree of correlation between the experimental and predicted values and there is more than 99% variation in response that could be predicted by second-order polynomial prediction equation. Adeq precision was reported to be 111.901 which indicates that the model is appropriate. The lack of fit *F*-value of 5.782 and *p*-value (*p* < 0.0001) was not at all valuable to the pure error and the fitness of the model was perfect. The high degree of precision and uniformity of the investigational outcomes were proved by the *p* value of lack of fit- 0.2830 (> 0.05) and a *p* value of probability (>F less than 0.05). The other linear and quadratic effects of glucose concentration, urea concentration, M-pH, and fermentation time were also significant (*p* < 0.0001). Finally, three-dimensional response surface plots and contour plots were constructed by Minitab (20.2) for a clear understanding of the effects of the parameters on GRH production (Figure 2).

**Table 3:** Experimental design and outcomes of the Box–Behnken Design (BBD) for optimisation of the GRH production from *Mucor* sp. HELF2.

Run	Independent variables				Response [GRH yield (g L^−1^)]	
					Measured	Predicted
	GC (x_1_)	PC (x_2_)	M-pH (x_3_)	FT (x_4_)		
1	−1(=10)	−1(=5)	0(=7.1)	0(=8)	21.189a_1_	21.602a_1_
2	1(=12)	−1	0	0	22.099a_2_	22.177a_2_
3	−1	1(=6)	0	0	21.984a_3_	22.067a_3_
4	1	1	0	0	21.684a_4_	21.433a_4_
5	0(=11)	0(=5.5)	−1(=6.9)	−1(=7)	22.083a_5_	22.162a_5_
6	0	0	1(=7.3)	−1	22.077a_6_	22.123a_6_
7	0	0	−1	1(=9)	21.733a_7_	21.848a_7_
8	0	0	1	1	21.779a_8_	21.862a_8_
9	−1	0	0	−1	22.139a_9_	22.032a_9_
10	1	0	0	−1	21.873a_10_	21.921a_10_
11	−1	0	0	1	21.789a_11_	21.663a_11_
12	1	0	0	1	21.686a_12_	21.715a_12_
13	0	−1	−1	0	22.206a_13_	22.066a_13_
14	0	1	−1	0	21.876a_14_	21.918a_14_
15	0	−1	1	0	22.164a_15_	22.044a_15_
16	0	1	1	0	21.853a_16_	21.915a_16_
17	−1	0	−1	0	22.114a_17_	21.975a_17_
18	1	0	−1	0	21.899a_18_	21.94a_18_
19	−1	0	1	0	22.083a_19_	21.957a_19_
20	1	0	1	0	21.88a_20_	21.933a_20_
21	0	−1	0	−1	22.307a_21_	22.199a_21_
22	0	1	0	−1	21.784a_22_	21.824a_22_
23	0	−1	0	1	21.801a_23_	21.675a_23_
24	0	1	0	1	21.749a_24_	21.772a_24_
25	0	0	0	0	22.57a_25_	22.583a_25_
26	0	0	0	0	22.621a_26_	22.583a_26_
27	0	0	0	0	22.583a_27_	22.583a_27_
28	0	0	0	0	22.581a_28_	22.583a_28_
29	0	0	0	0	22.562a_29_	22.583a_29_

One-way ANOVA (Tukey’s Multiple Comparison test) was performed to check the potential statistical differences (*P* < 0.05) between the measured and predicted GRH production. There were no statistical differences between each data set (row-wise) and similar letters (a_1_-a_29_) in each row indicate the data are the same and lack statistical differences.

**Table 4:** ANOVA for response surface quadratic regression model of Galactose Rich Heteropolysaccharide production by endophytic fungi *Mucor* sp. HELF2.

Source	DF	Sum of squares	Mean square	F-Value	*P*-Value prob.>F
Model	14	9.84399	0.19982	1994.082	<0.001
x_1_ (GC)	1	0.38702	0.58191	1082.069	<0.001
x_2_ (PC)	1	0.59729	0.81973	974.819	<0.001
x_3_ (M pH)	1	0.49871	0.80132	908.019	<0.001
x_4_ (FT)	1	0.29871	0.59713	801.011	<0.001
x^2^_1_ (GC*GC)	1	0.03152	0.57085	1109.039	<0.001
x^2^_2_ (PC*PC)	1	0.87199	0.87172	1339.760	<0.001
x^2^_3_ (M pH*M pH)	1	0.37189	0.57831	1039.93	<0.001
x^2^_4_ (FT*FT)	1	0.81923	0.79849	916.14	<0.001
x_1_ x_2_ (GC*PC)	1	0.39905	0.71280	919.82	<0.001
x_1_ x_3_ (GC*M pH)	1	0.41977	0.76862	808.015	<0.001
x_1_ x_4_ (GC*FT)	1	0.66890	0.92914	1139.19	<0.001
x_2_ x_3_ (PC*M pH)	1	0.90972	0.98132	996.02	<0.001
x_2_ x_4_ (PC*FT)	1	0.56491	0.89032	1138.091	<0.001
x_3_ x_4_ (M pH*FT)	1	0.69981	0.70237	767.091	<0.001
Residual	14	0.43781	0.20976		
Lack-of-Fit	10	0.53500	0.90493	5.782	0.2830
Pure Error	4	0.20778	0.96892		
Cor total	28	8.16903			
R^2^		0.999209			
Adj R^2^		0.999800			
Pred R^2^		0.995062			
Adeq precision		111.901			

The model predicted a maximum response of 20.10 g L^−1^ GRH yield when the necessary components are 5.2 g L^−1^ of peptone, 10.5 g L^−1^ of glucose, 7.05 MpH, and 180 (7.5 days) h of fermentation time. These predictions were authenticated by performing laboratory experiments in flask culture by triplicate with an outcome of 19.951 ± 0.091 g L^−1^ of GRH.

### Characterisation of the exopolysaccharide

**Figure 3:**
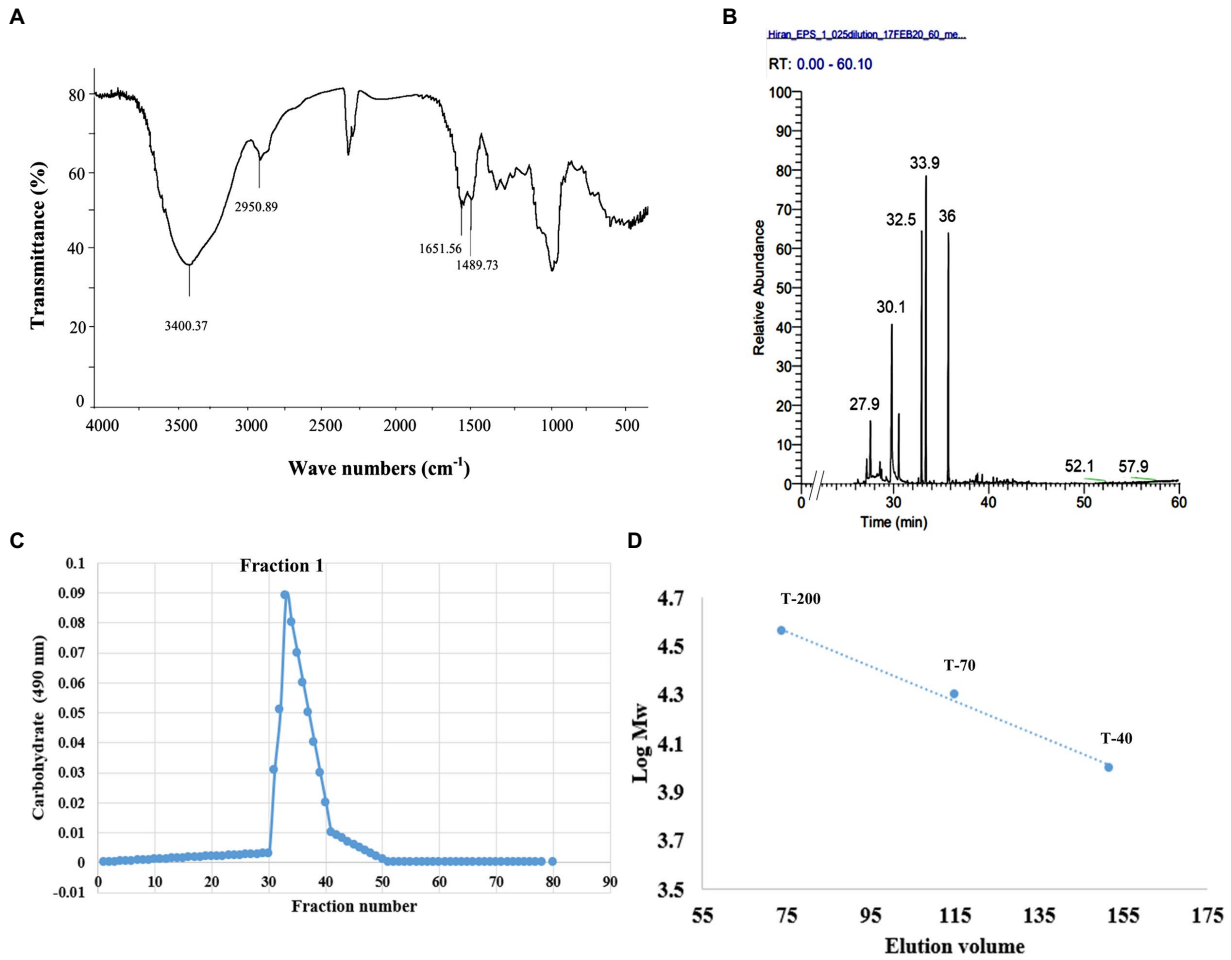
**(A)** FT-IR spectra of crude HELF2 GRH showing the necessary functional groups present in the sample. **(B)** GC–MS chromatogram of the derivatized fungal GRH showing the different peaks of monosaccharide compositions at different retention times. **(C)** Elution profile of the polysaccharide showing the occurrence of dominant fraction (F1) confirmed by carbohydrate test. **(D)** Standard curve of dextran needed for the detection of sugar concentration in the polysaccharide sample.

Exopolysaccharide produced by Endophytic *Mucor* sp. HELF2 was precipitated by applying chilled ethanol and crude EPS was then dialysed, and purified by gel filtration chromatography with a Sepharose-6B column. One major fraction obtained was eluted between 29 and 42 tubes (Figure 3C) and the colorimetric test confirms the absence of proteins in those fractions. The fraction was further investigated for monosaccharide analysis. The molecular weight of the homogeneous EPS was calculated from a calibration curve of standard dextran as ~2.98 × 10^5^ Da (Figure 3D). Monosaccharide analysis of the derivatised EPS samples showed the occurrence of galactose, fucose, and glucose in a 13:2:1 ratio with D, L, and D configuration, respectively (Table 5). Each repeating unit of the fraction contained 13 galactose, two fucoses, and one glucose, which indicates that the studied EPS contained approximately 104 repeating units. We, therefore, considered that the Galactose Rich Heteropolysaccharide (GRH) could have been produced by endophytic *Mucor* sp. HELF2. FT-IR analysis of the EPS sample revealed the occurrence of strong absorption peaks at particular wavelengths of 3400.71, 2950.89, 1651.56, 1489.73 which represents C-H, O-H, C-O asymmetric stretching respectively, which indicates the basic characteristics and purity of the carbohydrate moiety. Figures 3A,B represent the FT-IR spectrum and GC–MS spectrum of the crude and derivatised EPS, respectively.

**Table 5:** Monosaccharide units present in the Galactose Rich Heteropolysaccharide (synthesised by endophytic fungi *Mucor* sp. HELF2) are represented here with their respective sugar linkages and molar ratios.

Methylated sugars	Linkage types	Molar ratio	Major fragments (m/z)
2,3,4-Me_3_-Gal-	6)-Galp-(1-	5	40, 61, 72, 88, 103, 113, 131, 140, 159, 175, 191, 203, 219, 231
2,3-Me_2_-Galp	-4,6)-β-D-Galp-(1-	4	44,46,59,69,72,83,85,97,99, 119,127,129,141,157,161,187,200
3,4-Me_2_-Gal	-2,6)-α-D-Galp-(1-	4	44,72,89,100,131,157,173,189,233
2,3,4-Me_3_-Fuc	α-L-Fucp-(1-	2	44,72,89,103,113,117, 131,161,175
2,4-Me_2_-Glc	−3,6)-D-Glcp-(1-	1	39,43,58,74,88,99,100, 119,130,144,160,174,191,209,215,234,246

### Plant growth-promoting traits of the GRH

**Figure 4:**
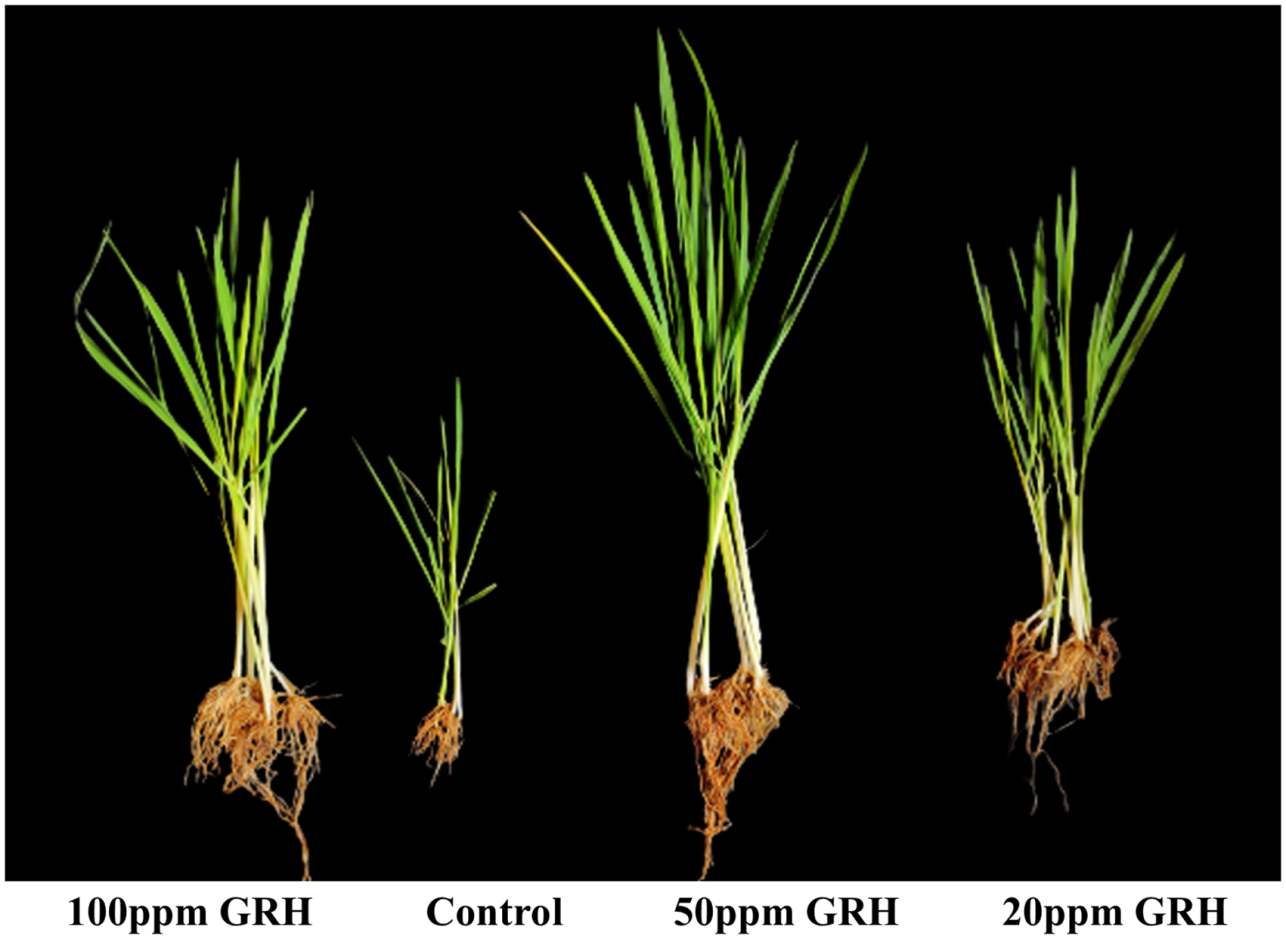
Phenotypes of rice seedlings *Oryza sativa* ssp. indica MTU 7093 Swarna under drought stress (induced by PEG treatment) sprayed with 20, 50, and 100 ppm EPS of *Mucor* sp. HELF2 endophyte.

GRH-sprayed rice seedlings were found to be much healthier, and more vigorous in terms of their fresh weight and relative water contents in comparison to the control (only drought-inducing agent-PEG treated). The control plants were characterised by low growth, chlorosis, and wilting of leaves. The rice seedlings exhibited maximum growth promotion after continuous 14 days of GRH treatment. The relative water content and fresh weight of the 50 ppm GRH treated plants were found to be higher than the plants of the control set, and plants treated with 20 ppm and 100 ppm GRH dosage. There was a 1.31, 2.38, and 1.74-time improvement in the fresh weight of seedlings in the 20, 50, and 100 ppm GRH treated plants compared to the control. The relative water contents were also increased by 1.14, 1.58, and 1.26 times in 20, 50, and 100 ppm GRH treated plants than the control one. There was a 3, and 4 times increase in root length and shoot length of the treated (50 ppm GRH) plant, respectively, compared to the control. The fresh weight of the seedlings were found to be improved after the GRH treatment and the 50-ppm GRH application was also found to be the most effective in comparison to the control. Table 6 represents the improved physical characteristics of the treated seedlings. Figure 4 represents the treated (20, 50, and 100 ppm) and control rice seedlings, showing their physical changes.

**Table 6:** Different physical parameters (fresh weight, root length, and shoot length) of GRH-treated and untreated drought-faced rice seedlings are represented here.

Treatment group (concentration of EPS)	Fresh weight (mg)	Root length (cm)	Shoot length (cm)	Relative water contents (%)
Control	97.81 ± 3.019a	2.01 ± 0.72a_1_	9.6 ± 1.73a_2_	69.02 ± 1.890a_3_
EPS 20 ppm	128.93 ± 4.051b	3.13 ± 0.69b_1_	13.36 ± 2.05b_2_	79.11 ± 1.472b_3_
EPS 50 ppm	232.93 ± 3.610c	6.61 ± 1.32c_1_	33.4 ± 3.28c_2_	109.31 ± 1.901c_3_
EPS 100 ppm	171.11 ± 2.098d	4.53 ± 0.81d_1_	23.41 ± 2.96d_2_	87.39 ± 1.014d_3_

One-way ANOVA (Tukey’s Multiple Comparison test) was performed to check the potential statistical differences and the treated (20, 50, and 100 ppm EPS foliar spray) plants showed statistically valid differences (*P* < 0.05, the four different letters for each of the four parameters (column-wise) indicates significance differences) from the control plant.

**Figure 5:**
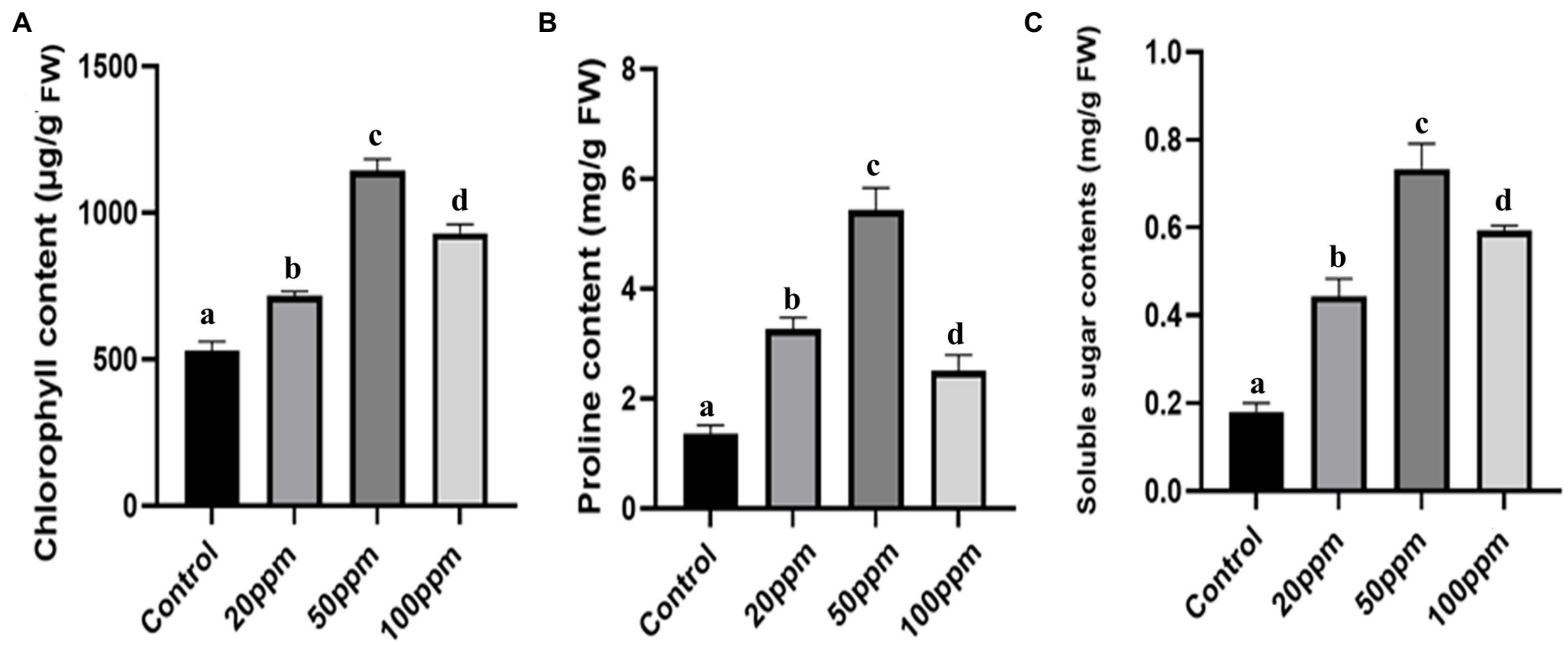
The effect of GRH foliar spray (20, 50, and 100 ppm) on chlorophyll content **(A)**, soluble sugar content **(B)**, proline content **(C)** of *O. sativa* ssp. indica MTU 7093 swarna in comparison to control. Values on the graphs are the means ± Standard error (SE) of three replicates. Tukey’s multiple comparison test was performed. The letters a, b, c, and d indicate significant differences compared to the control plant (At, *p* < 0.05).

**Figure 6:**
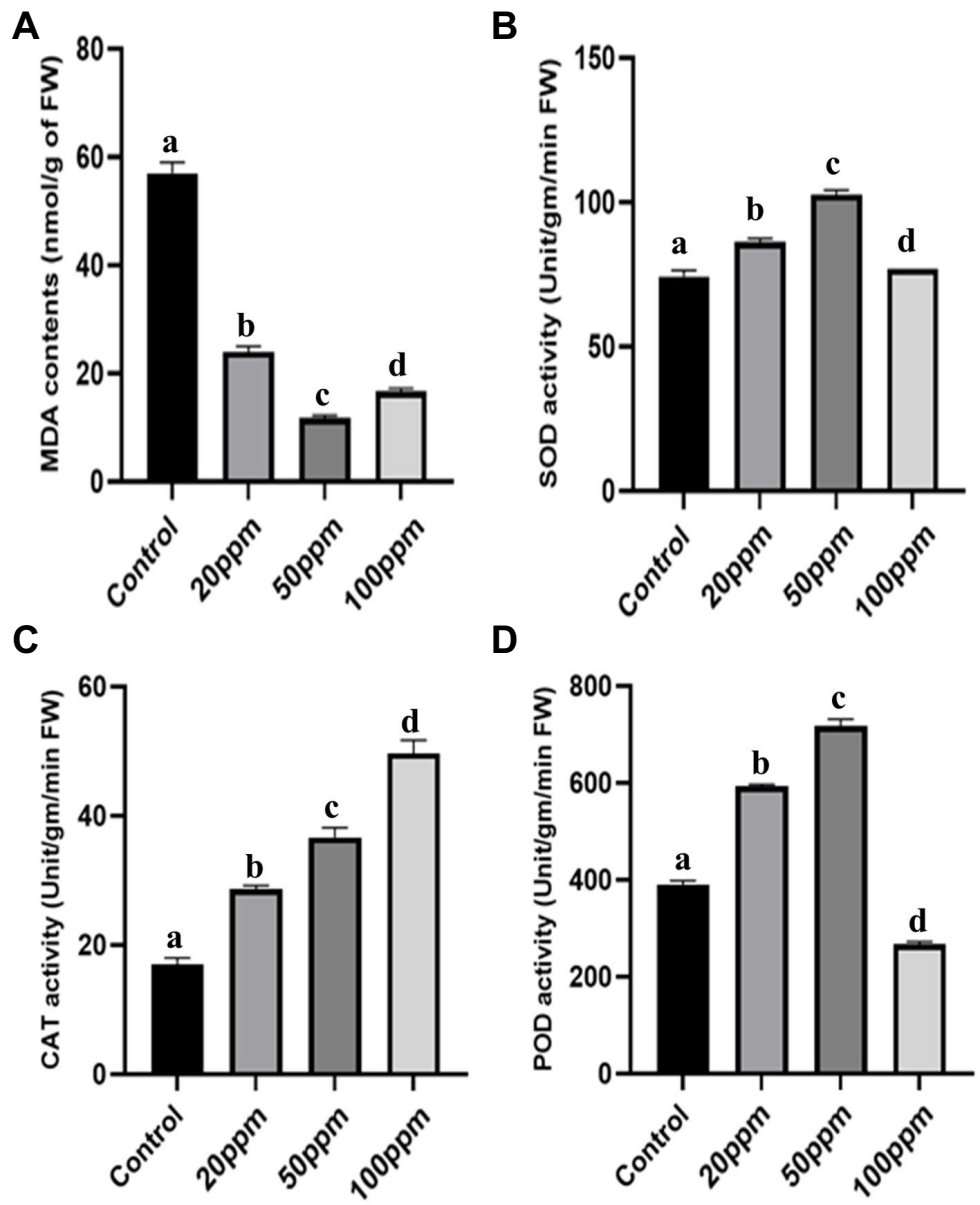
The effect of GRH foliar spray (20, 50, and 100 ppm) on malondialdehyde (MDA) contents **(A)**, peroxidase (POD) activity **(B)**, catalase (CAT) activity **(C)**, superoxide dismutase (SOD) activity **(D)** of *O. sativa* ssp. indica MTU 7093 swarna in comparison to control. Values on the graphs are the means ± Standard error (SE) of three replicates. Tukey’s multiple comparison test was performed. The letters a, b, c, and d indicate significant differences compared to the control plant (At, *p* < 0.05).

Not only physical but also biochemical characteristics were improved in the case of GRH-treated plants even after severe drought situations. The chlorophyll contents of the treated plants were found to be more elevated (2.32 times higher for 50 ppm GRH treated one) than the untreated control (Figure 5A). The proline is a potent indicator of plant stress and higher proline contents indicate higher resistance towards stress and better adaptation to that stressful situation. Here the GRH-treated plant shows higher accumulations (approximately 3.89 times higher for 50 ppm GRH treatment) of proline contents than the control ones (Figure 5B). The presence of increased soluble sugar content in the plant tissues also indicates the higher survival ability of plants in drought-stress situations. In this experiment, we found almost 3.5 times higher accumulation of soluble sugar contents in 50 ppm GRH-treated plants in comparison to the only PEG-treated one (Figure 5C). On the other hand, the MDA (Malondialdehyde) content is found to be correlated with lipid peroxidation and membrane damage. Higher MDA content in the plant tissues indicates the detrimental situation induced by the stress factors. In the case of treated seedlings, there was a sharp six time decrease in MDA contents compared to the control (Figure 6A). Other enzymatic antioxidative parameters were found to be also elevated after GRH treatment even in extreme drought situations. SOD, CAT, and POD activities increased up to 1.44, 2.09, and 1.79 times, respectively, in the case of GRH-treated rice seedlings compared to those treated only with PEG (Figures 6B–D).

## Discussion

Agriculture is seen as the most important and crucial sector of the global economy, and it significantly affects our GDP (Gross Domestic Production). The increased explosion in population in recent years has increased the demands for global agricultural output or food production by 60–100% by the end of 2050 to meet these growing needs, but the main obstacles are the lack of suitable fertile croplands and the rising instances of soil desertification due to insufficient precipitation, random evaporation, and a lack of freshwater resources, among other factors (Naumann et al., 2018; Dey et al., 2019; Paglia and Parker, 2021). Therefore, the primary requirement for a successful solution is the restoration of land or the development of salt or drought stress varieties. The development of drought-tolerant plants could temporarily meet the world’s food demand and protect crop plants, but the situation becomes severe when drought conditions (like the 2011–17 California drought and the 1997–99 Melbourne Millennium drought) occur on large scales around the globe. The food supply chain is hampered, and even the forest environment is impacted (Allen et al., 2010). Therefore, it is strongly recommended that deep ecological techniques that use non-toxic, natural substances be developed to address these issues. Exopolysaccharides produced by microbes, especially endophytes could have a significant impact (Chen et al., 2017). Even in situations with salt and drought challenges, endophytic fungi and bacteria are well known for their ability to promote plant growth (Azad and Kaminskyj, 2016; Bibi et al., 2019; Ali et al., 2021; Gupta et al., 2021). There are many reports on how microorganisms (both endophytes and rhizospheric) can reduce abiotic stress (Hammami et al., 2016), and endophytes often play an osmoprotective role in maintaining good water chemistry (managing Na^+^/K^+^ balance) within cells (Jha et al., 2011; Abdelaziz et al., 2017). Previous research has been undertaken, examining the role of endophytic fungi and bacteria in reducing the effects of salt stress in rice, maize, soybean, quinoa, barley, and barrel medic, as well as in the model plant *Arabidopsis thaliana* through endogenous hormone (abscisic acid) mediated morphological, biochemical (through ion balancing), and antioxidant defence-related pathways (Baltruschat et al., 2008; Bagheri et al., 2013; Jogawat et al., 2013; Li et al., 2017; Shahzad et al., 2017; Asaf et al., 2018; Fan et al., 2020; Ali et al., 2022; González-Teuber et al., 2022). Exo-polysaccharides and gamma-polyglutamic acid are also discovered to be the most useful compounds released by plant growth-promoting microbes and have exceptional biotic and abiotic stress tolerance (Livingston et al., 2009; Lei et al., 2017). Here, drought stress ameliorating properties of GRH was evaluated on rice plants. Due to their widespread popularity around the world, rice seedlings (*Oryza sativa* ssp. indica MTU 7093 Swarna- Indian subcontinental cultivar) were taken into consideration for their studies on drought relief. It is a very demanding staple food, especially in China, India, and Japan (Uga et al., 2013; Zhu, 2016), and has greater irrigation water needs (Bouman et al., 2005, 2007). According to recent studies, around forty-two million hectares of rice farming face significant challenges because of a lack of water. To formulate an appropriate response, we reported on how Galactose Rich Heteropolysaccharide reduces the effects of drought stress on rice plants. Chen et al. (2017) and Santra and Banerjee (2022a) both found that the application of direct endophyte and EPS produced from endophyte alleviated salt and drought stress in wheat and rice plants, respectively. Due to their high polymeric configurations, effective water-holding capacities, and strong affinity to create bio-films or similar sorts of aggregations, polysaccharides are thought to have significant crop resistance (against both biotic and abiotic) enhancers and plant growth promotors (Muley et al., 2019). Chitosan, β-D-glucan, and other microbial polysaccharides have been found to have growth-stimulating and systemic disease resistance-inducing characteristics on cash crops such as *Solanum lycopersicum*, *Hordeum vulgare*, *Solanum tuberosum*, *Saccharum officinarum*, *Gossypium herbaceum*, and *Glycine max* (Uyen, 2014; Gandra et al., 2016; Blainski et al., 2018; Zhang et al., 2020).

The physical and biochemical traits of seedlings treated with GRH significantly improved. Proline levels and soluble sugar characteristics were also found to be altered, improving the stress-tolerating enzyme profile and enabling the plant to adapt to dry circumstances more successfully. The lower levels of MDA suggest that lipid peroxidation has significantly decreased and that membrane damage has become less frequent. The best concentration of EPS (i.e., Galactose rich heteropolysaccharide- GRH) for controlling the stressful condition was 50 ppm. Lower treatment doses are not sufficient to cause a noticeable change in the plantlets, whereas greater concentrations of GRH are likely to have a negative effect on the health of the plant. The findings of Sun et al. (2020b) are consistent with our findings because the 50 ppm EPS application was also determined to be the best-fitting one in that instance. Through a rise in endogenous ABA levels, polysaccharide treatments affect the stomatal physiology of the test plants’ leaves and cause partial stomatal closure, which minimises water evaporation. To improve the internal water levels of the tissues, which are essential for the plant’s proper growth and metabolism, these bioactive compounds function as anti-transpirant agents (Vishwakarma et al., 2017). Treatments with GRH increase the relative water contents of the tissues in this instance as well. The uniform build-up of rigid and highly water-soluble osmolytes (such as sugars, amino acids, and prolines) throughout the plant tissues, which provides subcellular stability and integrity, is another mechanism by which exopolysaccharide-mediated drought stress relief works (Hare et al., 1998; Krishnan et al., 2008). Increased levels of osmolyte aggregation raise osmotic pressure, which in turn induces higher water intake and insignificant water outflow. This keeps the cells’ critical osmotic pressure constant needed for optimum cell growth and division (Kaur and Asthir, 2015). The proline and soluble sugar levels (osmolytes) are increased by 50 ppm of GRH treatment in the current study as well, balancing the ideal subcellular environment for a healthy water weight required for cell growth. Proline is found to be an important osmo-regulator, and its exogenous administration increases hosts’ resistance to abiotic stress (Yoshiba et al., 1997; Ben Ahmed et al., 2010). For abiotically challenged plants, exogenous administration of water-soluble polysaccharides also demonstrates a similar response and causes a significant rise in proline levels (Yu et al., 2017; Zou et al., 2018). Thus, in this instance, GRH functions as a biological elicitor or priming agent that activates the cascades of biochemical processes required for water balance and antioxidant defence—ROS scavenging. Thus, as seen in the cases of rice, parsley, and tobacco, polysaccharides generate faster activation of transcription factors leading to the expression of defence-related genes, increasing the alleviation of drought stress (Conrath et al., 2002, Ortmann et al., 2006, Bozsoki et al., 2017). In drought-stricken areas, microbial exopolysaccharide enhances plant development by up- and down-regulating the expression of proline synthase and proline dehydrogenase, respectively (Sun et al., 2020a). By increasing SOD, POD, and CAT levels and fostering the effective operation of cellular biochemical machinery, which is essential for the survival of the plant, osmolytes also effectively eliminate harmful free radicals (reactive oxygen species) under drought stress (Sun et al., 2020b). The three key members of the antioxidant system SOD, CAT, and POD act in an integrated approach, where SOD acts as the first line of defence and converts superoxide free radicals to H_2_O_2_, which is further catalysed into water and oxygen by CAT and POD (Das and Roychoudhury, 2014). Last but not least, the MDA concentrations decrease, reducing the peroxidation of membrane proteins and lipids (Fu et al., 2010; Miller et al., 2010). Spraying potato and wheat with chitosan, polysaccharides from *Ganoderma lucidum*, *Lactobacillus plantarum*, and *Pantoea agglomerans*, respectively, activates the antioxidant defence cascades (Ortmann et al., 2006; Blainski et al., 2018; Muley et al., 2019; Zhang et al., 2020). Here, foliar GRH spray applied at a dosage of 50 ppm enhanced the SOD, POD, and CAT levels while concurrently lowering the MDA contents.

The bio-active GRH produced by *Mucor* sp. HELF2 was a polymer of D-galactose, L-fucose, and D-glucose (molar ratio—13:2:1) with a molecular weight of 2.98 × 10^5^ Da. Galacto-rhamnan and beta-glucan exopolysaccharides with molecular weights of 1.87 × 10^5^ and 2 × 10^5^ Da were found in endophytic *Fusarium* sp. SD5 and *Pestalotiopsis* sp. BC55, respectively, according to Mahapatra and Banerjee’s reports from 2013 and 2016. Polysaccharides from edible mushroom *Termitomyces heimii*, and *Meripilus giganteus* also represents a similar type of monosaccharide compositions of L-fucose, D-galactose, and D-glucose, etc. (Maity et al., 2017, 2020). The EPS manufacturing process was optimised for carbon, nitrogen sources, a medium pH, and fermentation temperature to produce the greatest quantity of polysaccharides. The appropriate oxygen demand was also considered. The optimisation data makes it possible to quickly and affordably obtain the polysaccharides in large quantities. The results of the current inquiry on the optimization of GRH production broadly concur with those of Mahapatra and Banerjee (2013, 2016).

The present study examined endophytic exopolysaccharides (GRH) from an ecologically valuable plant and checked the drought tolerance action of the GRH on rice plants. Finally, varying concentrations of fungal EPS were used to reduce the effects of drought stress. Our research clarifies the idea of creating rice types resistant to drought through the external application of EPS, which supports environmentally friendly farming methods. This study provides the first evidence of the use of endophytic *Mucor* sp. HELF2 produced D-galactose-rich heteropolysaccharide to reduce drought stress in rice seedlings.

## Conclusion

In the present study, rice seedling dehydration stress was lessened by a galactose-rich heteropolysaccharide derived from endophytic *Mucor* sp. HELF2. The outcome illustrated that treated plants had higher fresh weights, relative water contents, and chlorophyll levels. While the MDA concentration reduced, osmolytes such as soluble sugars, proline, as well as the antioxidant defence enzymes SOD, CAT, and POD, increased. The results support the conclusion that foliar spray of Galactose Rich Heteropolysaccharide efficiently promotes drought resistance in rice plants. GRH production was also optimised by adopting OVAT and RSM techniques and there was a 1.5-fold (20.10 g L^−1^) enhancement in GRH production in optimised fermentation conditions. The ability of GRH to alleviate the effects of drought stress on rice plants and the high yield of GRH makes it suitable for commercial exploitation. The current investigation’s findings may encourage sustainable farming methods and have an impact on the cultivation of crops in drought-prone areas.

## Data availability statement

The datasets presented in this study can be found in online repositories. The names of the repository/repositories and accession number(s) can be found at: https://www.ncbi.nlm.nih.gov/genbank/, ON146358.

## Author contributions

HS designed and performed the experiments, and prepared the draft of the manuscript. DB designed the experiment and finalised the manuscript. All authors contributed to the article and approved the submitted version.

## Conflict of interest

The authors declare that the research was conducted in the absence of any commercial or financial relationships that could be construed as a potential conflict of interest.

## Publisher’s note

All claims expressed in this article are solely those of the authors and do not necessarily represent those of their affiliated organizations, or those of the publisher, the editors and the reviewers. Any product that may be evaluated in this article, or claim that may be made by its manufacturer, is not guaranteed or endorsed by the publisher.
